# Increased aortic stiffness and blood pressure in non-classic Pompe disease

**DOI:** 10.1007/s10545-013-9667-2

**Published:** 2014-01-10

**Authors:** Stephan C. A. Wens, Esther Kuperus, Francesco U. S. Mattace-Raso, Michelle E. Kruijshaar, Esther Brusse, Kees C. A. G. M. van Montfort, Marjan Scheltens- de Boer, Eric J. G. Sijbrands, Ans T. van der Ploeg, Pieter A. van Doorn

**Affiliations:** 1 Department of Neurology, Erasmus University Medical Center, Mailbox 2040, 3000 CA Rotterdam, The Netherlands; 2 Center for Lysosomal and Metabolic Diseases, Erasmus MC, Rotterdam, The Netherlands; 3 Department of Internal Medicine, Erasmus MC, Rotterdam, The Netherlands; 4 Department of Pediatrics, Division of Metabolic Diseases and Genetics, Erasmus MC-Sophia, Rotterdam, The Netherlands; 5 Department of Epidemiology and Biostatistics, Erasmus MC, Rotterdam, The Netherlands

## Abstract

Vascular abnormalities and glycogen accumulation in vascular smooth muscle fibres have been described in Pompe disease. Using carotid-femoral pulse wave velocity (cfPWV), the gold standard methodology for determining aortic stiffness, we studied whether aortic stiffness is increased in patients with Pompe disease. Eighty-four adult Pompe patients and 179 age- and gender-matched volunteers participated in this cross-sectional case-controlled study. Intima media thickness and the distensibility of the right common carotid artery were measured using a Duplex scanner. Aortic augmentation index, central pulse pressure, aortic reflexion time and cfPWV were assessed using the SphygmoCor® system. CfPWV was higher in patients than in volunteers (8.8 versus 7.4 m/s, *p* < 0.001). This difference was still present after adjustment for age, gender, mean arterial blood pressure (MAP), heart rate and diabetes mellitus (*p* = 0.001), and was shown by subgroup analysis to apply to the 40-59 years age group (*p* = 0.004) and 60+ years age group (*p* = 0.01), but not to younger age groups (*p* = 0.99). Except for a shorter aortic reflexion time (*p* = 0.02), indirect indicators of arterial stiffness did not differ between patients and volunteers. Relative to volunteers (20 %), more Pompe patients had a history of hypertension (36 %, *p* = 0.005), and the MAP was higher than in volunteers (100 versus 92 mmHg, *p* < 0.001). This study shows that patients with non-classic Pompe disease have increased aortic stiffness and blood pressure. Whether this is due to glycogen accumulation requires further investigation. To reduce the potential risk of cardiovascular diseases, we recommend that blood pressure and other common cardiovascular risk factors are monitored regularly.

## Introduction

Pompe disease (OMIM 232300: acid maltase deficiency or glycogen storage disease type II) is an inheritable lysosomal storage disorder caused by a deficiency of acid α-glucosidase that leads to glycogen accumulation in various body tissues, predominantly skeletal, cardiac and smooth muscle (Hirschhorn 2001; van der Ploeg and Reuser 2008). Patients with the classic-infantile form of the disease develop generalized hypotonia and a hypertrophic cardiomyopathy. Without treatment, these patients die in the first year of life due to cardiorespiratory failure (van den Hout et al 2003). In the non-classic form of the disease, which can present at any age, progressive muscle weakness is the predominant symptom. In general, there is no significant cardiac involvement (Soliman et al 2008; van der Beek et al 2012).

Over the last two decades, several reports have hypothesized that glycogen accumulation in the smooth muscle tissue of arteries leads to vascular abnormalities in non-classic Pompe disease, such as cerebral aneurysms, basilar artery dolichoectasia, carotid dissection and dilated arteriopathy of the thoracic aorta (Braunsdorf 1987; Winkel et al 2003, 2005; Laforet et al 2008; Sacconi et al 2010; El-Gharbawy et al 2011). The first indication of increased aortic stiffness in non-classic Pompe disease was reported in a study on 17 Pompe patients that used transthoracic Doppler echocardiography, a non-invasive tool that measures aortic stiffness indirectly (Nemes et al 2007). The presence of increased aortic stiffness may be relevant, since it is considered to be an independent risk factor for cardiovascular disease and mortality (Mattace-Raso et al 2006; Verbeke et al 2011). The emerging gold standard for measuring it directly and non-invasively is tonometry of the carotid-femoral pulse wave velocity (cfPWV) (Van Bortel et al 2012).

To determine whether increased arterial stiffness is a feature of non-classic Pompe disease, we used various techniques including tonometry of the cfPWV to investigate the structural and functional vascular properties of different vascular territories in a large group of patients with non-classic Pompe disease. We compared the results with those in age- and gender-matched volunteers.

## Materials and methods

### Study population

Between March 2012 and June 2013 we performed a cross-sectional single-centre case-controlled study in 84 patients with non-classic Pompe disease and 179 volunteers. All patients were 18 years or older. As the volunteers were matched for gender and age category, there were, per patient, at least two volunteers of the same gender and the same age category. The volunteers were either the patients’ partners, or were employees or students of Erasmus University Medical Center, or otherwise visitors to it. Informed consent was obtained from all participants. The study protocol was approved by the Medical Ethical Committee at Erasmus University Medical Center

### Cardiovascular risk factors

All participants filled out a questionnaire to assess cardiovascular risk factors, which included gender, date of birth, length, weight, medication use, and comorbidities such as hypertension, diabetes mellitus, hypercholesterolemia and cardiovascular disease (i.e. transient ischemic attack, stroke, myocardial infarction or rhythm disturbances). Body mass index (BMI), which was calculated as body weight divided by height squared, was expressed as kg/m^2^. An automatic mercury cuff sphygmomanometer on the right arm was used to measure heart rate and blood pressure, including the mean arterial blood pressure (MAP), when the patient was in supine position after 5 min of rest in a quiet and heat-controlled room. Hypertension was defined as a systolic blood pressure (SBP) > 140 mmHg and/or diastolic blood pressure (DBP) > 90 mmHg (Mancia et al 2013).

### Vascular measurements

Aortic stiffness was measured using arterial tonometry from the right radial, right carotid and right femoral arteries using the SphygmoCor**®** device (Sphygmocor version 7.1, AtCor Medical, Sydney, Australia) according to previously described procedures (van Dijk et al 2013). Transit distance was assessed on the basis of body surface measurement from the carotid artery to the femoral artery: 80 % of this distance was used as pulse-wave-travelled distance (Van Bortel et al 2012). The primary outcome measure, cfPWV, was expressed in meters per second (m/s). The secondary and indirect outcome parameters for arterial stiffness measured with pulse wave analysis (PWA) of the right radial artery were central pulse pressure (PPc), augmentation index (AIx) and aortic reflexion time (ATr). PPc was calculated as the difference between aortic systolic and diastolic pressure; AIx was calculated from the aortic pressure waveform obtained by applying a general transfer function to the radial pressure waveform. For the comparison between two groups, AIx was normalized to a heart rate of 75 beats per minute. ATr was calculated as the time interval between the foot of the pressure wave and the shoulder of the reflected wave (Laurent et al 2006; DeLoach and Townsend 2008; Huijben et al 2012).

The distensibility and IMT of the right common carotid artery were measured two centimetres below the carotid bifurcation using a vessel-wall movement-detector system (Art.Lab, Esaote Europe, Maastricht, the Netherlands) according to previously described procedures (van Dijk et al 2013). The distensibility coefficient, a local measurement of carotid stiffness, was calculated as (2ΔD/D)/PP(10^−3^/kPa) (Hoeks et al 1992), in which D is the diameter during diastole, ΔD is the measured distensibility (systolic diameter – diastolic diameter), and PP is the aortic pulse pressure derived from radial applanation. Tonometry and ultrasonography were performed by two trained physicians (SW or EK).

### Statistical analyses

Unless otherwise specified, data are presented as medians with interquartile ranges (IQR). Because cfPWV, PPc, AIx, ATr, distensibility and IMT were not normally distributed, the Mann–Whitney test was used to compare these outcomes between the total groups of patients and volunteers.

Linear regression analyses were used to study cfPWV separately. First, univariate regression analyses were performed. Next, the regression coefficients with a p-value less than 0.05 or variables that were considered clinically relevant were analysed in a multivariate regression model using bootstrap estimation. The final multivariate model contained age, gender, MAP, heart rate and diabetes mellitus.

We also performed a stratified analysis between patients and volunteers in three age groups: 20-39, 40-59 and 60+. To determine whether cfPWV was influenced by disease duration, treatment with ERT, and wheelchair or ventilator dependency, we also analysed cfPWV in separate multivariate regression models using bootstrap estimation. The final model for these analyses included four variables: age, gender, MAP and heart rate. The data were analysed using SPSS 20. A p-value of less than 0.05 was considered statistically significant.

## Results

### Study population

Table 1 shows the characteristics of the two groups. In total, 84 patients with non-classic Pompe disease and 179 volunteers were included in the study. Twenty-six percent of the patients were wheelchair dependent, and 31 % needed artificial ventilation. Eighty-two percent of the patients were treated with ERT and the median duration of treatment was 5 years (IQR 4-5). CfPWV could not be measured in nine patients, either because they could not lie in supine position due to respiratory failure, or because their use of accessory muscles made it difficult to visualize the carotid artery. Neither could cfPWV be measured in the other eight volunteers due to temporary technical problems with the SphygmoCor® system.

**Table 1:** Demographic and clinical characteristics

	Pompe patients	Volunteers
Number of individuals	84	179
Age in years	54 (42-63)	54 (41-61)
Gender – male (%)	41 (49)	85 (48)
Body mass index in kg/m^2^	24.7 (22.0-27.3)	24.0 (22.2-26.0)
Smoking status (%)		
▪ Never	36 (43)	107 (60)
▪ Past	41 (49)	53 (29)
▪ Current	7 (8)	19 (11)
Medical history		
▪ Hypertension (%)	30 (36)^a^	35 (20)
▪ Diabetes mellitus (%)	5 (6)	6 (3)
▪ Hypercholesterolemia (%)	8 (10)	18 (10)
▪ Cardiovascular disease (%)	7 (8)	9 (5)
Antihypertensive medication (%)	23 (77)	31 (89)
Disease duration in years	16 (12-23)	-
Patients receiving ERT (%)	69 (82)	-
Duration of ERT in years	5 (4-5)	-
Wheelchair use (%)	22 (26)	0 (0)
Ventilator use (%)	26 (31)	0 (0)

Continuous variables are given as medians with interquartile ranges (IQR), and categorical variables as numbers and percentages

^a^In the past, more patients with Pompe disease than volunteers were diagnosed with hypertension (p-0.005)

### Cardiovascular risk factors

In the past, more patients with Pompe disease than volunteers had been diagnosed with hypertension (*p* = 0.005, Table 1). Seventy-seven per cent of the Pompe patients with an earlier diagnosis of hypertension used antihypertensive medication at the time of evaluation, and 89 % in the group of volunteers. Despite treatment with antihypertensive medication, blood pressure continued to be high in 52 % of the Pompe patients who were treated for hypertension and in 65 % of the volunteers. As Table 2 shows, MAP was higher in patients than in volunteers (100 mmHg (IQR 90-111) versus 92 mmHg (IQR 84-103), *p* < 0.001). Thirty-nine per cent of the patients met the criteria for hypertension (SBP > 140 mmHg and/or DBP > 90 mmHg), versus 20 % of the volunteers (*p* = 0.001). At group level, patients with Pompe disease had a higher heart rate (75 beats per minute (bpm) (IQR 68-83)) than the volunteers (65 bpm (IQR 59-71), *p* < 0.001).

**Table 2:** Functional and structural hemodynamic parameters in patients and volunteers

	Pompe patients (*n* = 84)	Volunteers (*n* = 179)	*P*-value
Mean arterial pressure (mmHg)	100 (90-111)	92 (84-103)	<0.001
Heart rate (beats per minute)	75 (68-83)	65 (59-71)	<0.001
Diameter carotid artery (mm)	6.6 (6.1-7.3)	7.1 (6.5-7.8)	<0.001
Intima media thickness (μm)	582 (514-683)	592 (506-695)	0.68
Distensibility coefficient (10^−3^/kPa)	2.9 (1.9-4.1)	2.7 (2.2-4.1)	0.72
Carotid-femoral pulse wave velocity (m/s)	8.8 (7.1-10.7)	7.4 (6.6-8.6)	<0.001
Central pulse pressure (mmHg)	38 (31-47)	36 (31-43)	0.30
Augmentation index (%)	21.7 (13.2-30.3)	19.4 (8.5-29.4)	0.23
Aortic reflexion time (ms)	140 (132-149)	144 (135-159)	0.02

Continuous variables are given as medians with interquartile ranges (IQR). The diameter of the carotid artery was measured in 179 volunteers and 84 patients; IMT was measured in 179 volunteers and 83 patients; the distensibility coefficient was measured in 176 volunteers and 80 patients; cfPWV was measured in 171 volunteers and 79 patients; and PPc, Aix and ATr were measured in 178 volunteers and 83 patients

There were no differences in the occurrence of comorbidities such as diabetes mellitus or hypercholesterolemia. Seven patients with Pompe disease had a medical history of cardiovascular disease: four of them (three female) had had a transient ischemic attack between the ages of 48 and 62 years); two male patients had developed atrial fibrillation at the ages of 37 and 64 years; and one female patient had had a myocardial infarction at the age of 74 years. Nine volunteers had a medical history of cardiovascular disease. This was not statistically different from the seven Pompe patients (*p* = 0.30).

### Aortic stiffness

**Fig. 1:**
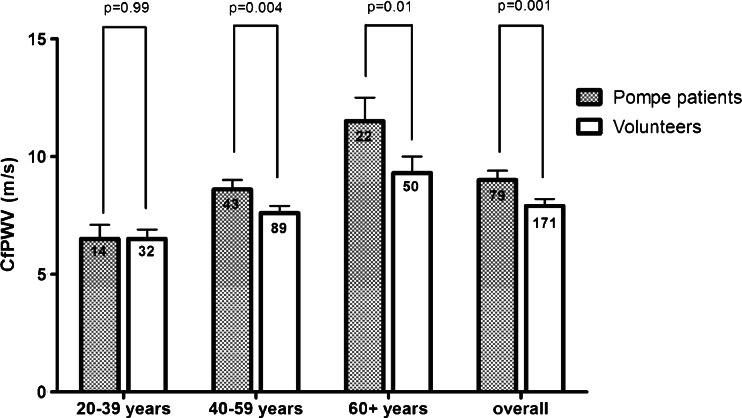
Mean values of cfPWV in patients and volunteers per age category. The *boxplots* represent the mean values and 95 % CI of cfPWV per age category after adjustment for age, gender, mean arterial blood pressure, heart rate and diabetes mellitus. The number of patients and volunteers per age group are given in the *bars*

As shown in Table 2, median cfPWV was 8.8 m/s in patients with Pompe disease (IQR 7.1-10.7) and 7.4 m/s in volunteers (IQR 6.6-8.6) (*p* < 0.001). This difference was still present after adjustment for age, gender, MAP, heart rate and diabetes mellitus (*p* = 0.001). Subgroup analysis showed that while it occurred in the 40-59 years age group (8.6 m/s in patients versus 7.6 m/s in volunteers, *p* = 0.004) and the 60+ years (11.5 versus 9.3 m/s, *p* = 0.01), it did not occur in the 20-39 years age group (Fig. 1). PPc and AIx did not differ significantly between patients and volunteers, while ATr was shorter in patients than in volunteers (*p* = 0.02).

CfPWV did not differ between Pompe patients whose disease duration was shorter than 15 years (*n* = 35) and those whose disease duration was longer than 15 years (*n* = 44) (9.1 versus 9.4 m/s, *p* = 0.51). Neither did it differ between patients treated with ERT (*n* = 64) and treatment-naïve patients (*n* = 15) (9.4 versus 8.6 m/s, *p* = 0.17). Similarly, cfPWV did not differ between wheelchair-dependent patients (*n* = 12) and ambulant patients (*n* = 62) (9.4 versus 9.2 m/s, *p* = 0.75), or between ventilator-dependent patients (*n* = 18) and non-ventilated patients (*n* = 56) (9.6 versus 9.1 m/s, *p* = 0.42).

### Carotid stiffness and IMT

Table 2 shows that there were no significant differences in distensibility or IMT between patients and volunteers. The diameter of the carotid artery was smaller in patients than in volunteers (6.6 mm (IQR 6.1-7.3) versus 7.1 mm (IQR 6.5-7.8), *p* < 0.001). Subgroup analysis showed that the distensibility was lower in patients with Pompe disease aged between 20 and 39 years than in volunteers (*p* = 0.03), but not in the other age-related subgroups.

### Glycogen accumulation in vascular smooth muscle

**Fig. 2:**
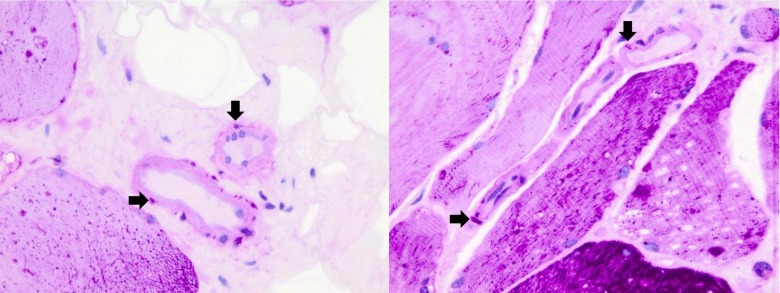
Glycogen accumulation in vascular smooth muscle fibres. Muscle biopsies of adult patients with non-classic Pompe disease stained with periodic acid-Schiff (PAS) to demonstrate glycogen storage in vascular smooth muscle fibres (*black arrowheads*). Original magnification: 630×

Figure 2 shows the muscle biopsy of a 50-year-old male patient with Pompe disease who participated in this study and had no medical history of cardiovascular diseases. He developed the first symptoms at age 44, and now has severe limb-girdle weakness and decreased pulmonary function in sitting and supine positions. The biopsy was stained with periodic acid-Schiff (PAS) and shows glycogen storage in skeletal muscle fibres and in vascular smooth muscle fibres.

## Discussion

We found that patients with the non-classic form of Pompe disease have increased aortic stiffness and blood pressure. These findings indicate clinical relevant involvement of the cardiovascular musculature in this group of Pompe patients.

Glycogen accumulation in smooth muscle fibres and in the endothelial layer of arteries has been shown in morphological studies and autopsy reports of patients with Pompe disease (Winkel et al 2003; Thurberg et al 2006; Kobayashi et al 2010). Figure 2 shows glycogen storage in vascular smooth muscle fibres in one of our adult patients with Pompe disease before the start of ERT. While it is likely that the increased glycogen storage in smooth muscle fibres results in an increased arterial stiffness, it is also possible that glycogen accumulation in the endothelium damages the vascular wall, making it more vulnerable to atherosclerosis; in the presence of other cardiovascular risk factors, this process might increase arterial stiffness. As vascular stiffness has also been described as depending on the distribution of collagen and elastin, an increase in abnormal collagen and a decrease in normal elastin contributes to greater arterial stiffness (Laurent et al 2006). During the course of the disease, patients with Pompe disease may develop striated muscle atrophy with some increase in connective tissue. If this process also occurred in the vascular smooth muscle fibres of Pompe patients, it would help to explain increased aortic stiffness.

Increased aortic stiffness has also been described in other lysosomal storage disorders such as Fabry disease and mucopolysaccharidosis type I (MPS I or Scheie syndrome). In Fabry disease, storage of glycosphingolipids takes place in vascular endothelial and smooth muscle cells, and in MPS I, accumulation of glycosaminoglycans in the connective tissue of the aortic wall impairs the formation of elastin and leads to changes in collagen formation (Nemes et al 2008; Collin et al 2012). It has been shown in Fabry disease that ERT can normalize aortic stiffness (Collin et al 2012). As most of the patients with Pompe disease enrolled in our cross-sectional study were treated with ERT, the difference in cfPWV between patients and volunteers might have been even more pronounced if it had been measured before the start of ERT. It would thus be interesting to establish whether aortic stiffness in Pompe patients decreases during ERT, and whether cfPWV can be used as a non-invasive tissue biomarker for disease progression and therapy response.

To the best of our knowledge, this is the first study to show that arterial blood pressure is increased in patients with Pompe disease. It is reasonable to assume that any structural changes in the blood vessels caused by the increased aortic stiffness would lead to higher blood pressure. Increased aortic stiffness and hypertension are both independent risk factors for cardiovascular disease and mortality (Mattace-Raso et al 2006; Verbeke et al 2011). It has been shown that patients with non-classic Pompe disease have improved survival when treated with ERT (Güngör et al 2013). Since cardiovascular diseases are more common at a higher age, Pompe patients will be more likely to develop cardiovascular diseases, as they owe their higher age to ERT. In these patients it is therefore important to closely monitor risk factors for cardiovascular diseases such as hypertension, hypercholesterolemia, diabetes mellitus and smoking, and to treat them when indicated. Although exercise and training, dietary changes and pharmacological treatments have sometimes been shown to reduce arterial stiffness in healthy individuals and other patient groups (Kingwell et al 1997; Girerd et al 1998; Balkestein et al 1999), it has not yet been investigated whether they are also beneficial in Pompe patients.

Although we expected Pompe disease to have a similar effect on the vascular structure of all arteries, and thereby to increase cfPWV and reduce distensibility, we found changes in carotid distensibility only in patients aged between 20 and 39 years. In those with high blood pressure and diabetes it has been reported that the aorta stiffened more than the carotid artery with age and with other cardiovascular risk factors such as increased body mass index and heart rate (Paini et al 2006). These findings may have been paralleled by our own finding in most of the Pompe patients whose blood pressure and heart rate were increased, who also had greater aortic stiffness without decreased distensibility.

A strength of our study is that we used cfPWV, which is currently the gold standard for measuring aortic stiffness directly and non-invasively in a relatively large group of Pompe patients. A previous study that found increased aortic stiffness in a small group of patients with Pompe disease used transthoracic echocardiography, an indirect way of measuring aortic stiffness (Nemes et al 2007).

Our study has some limitations. First, due to physical or temporary technical problems, measurements of aortic stiffness were not available for all subjects. However, as most of the patient data that were missing in the group of Pompe patients were those for the elderly and more severely affected patients, this would probably have led to an underestimation of the real difference in aortic stiffness between Pompe patients and healthy volunteers. Second, as this was a cross-sectional study, we do not know about the progression of aortic stiffness over time, or about the effects of ERT on aortic stiffness. Additionally, rather than measuring glucose or cholesterol in blood samples, we used a questionnaire to establish whether patients had a history of hypercholesterolemia or diabetes mellitus.

In conclusion, patients with non-classic Pompe disease have increased aortic stiffness and blood pressure. To reduce the potential risk of cardiovascular diseases, we recommend that blood pressure and other common cardiovascular risk factors are monitored closely. Prospective studies should investigate whether ERT reduces aortic stiffness, or whether early treatment even prevents it.

## Abbreviations

AIx: augmentation index
ATr: aortic reflexion time
CfPWV: carotid-femoral pulse wave velocity
DBP: diastolic blood pressure
ERT: enzyme replacement therapy
IMT: intima media thickness
MAP: mean arterial blood pressure
PAS: periodic acid-Schiff
PPc: central pulse pressure
PWA: pulse wave analysis
SBP: systolic blood pressure
